# The carotid axis revisited

**DOI:** 10.1038/s41598-021-93397-0

**Published:** 2021-07-05

**Authors:** R. Cobiella, S. Quinones, M. Konschake, P. Aragones, X. León, T. Vazquez, J. Sanudo, E. Maranillo

**Affiliations:** 1 Department of Anatomy and Embryology, School of Medicine, Complutense University of Madrid, Madrid, Spain; 2 Department of Anatomy, Histology and Embryology, Institute of Clinical and Functional Anatomy, Medical University of Innsbruck (MUI), Müllerstr. 59, 6020 Innsbruck, Austria; 3 Department of Orthopedics. Hospital, Universitario Santa Cristina, Madrid, Spain; 4 Department of ORL, Hospital de La Santa Creu I Sant Pau, UAB, Barcelona, Spain

**Keywords:** Preclinical research, Translational research

## Abstract

The aim was to determine the variations in the level of origin of carotid bifurcation and diameters of the common, internal, and external carotid arteries which is clinically important for several interventional procedures. Therefore, 165 human embalmed corpses were dissected. The data collected were analyzed using the Chi square-test and the Pearson correlation test. The results of previous studies have been reviewed. In relation to the level of the carotid bifurcation, taking as a reference point the hyoid bone, the values ranged from 4 cm below the hyoid body to 2.5 cm above the body of the hyoid, being the average height—0.33 cm, with a standard deviation of 1.19 cm. The right carotid bifurcation was established at a higher level (x = − 0.19 cm.) than the left one (x = − 0.48 cm.) (p = 0.046). On the contrary, no significant gender differences could be observed. The arterial calibres of the common and internal carotid arteries were higher in male than female. In the internal carotid artery (X = 0.76 cm.), the left was greater than the right (X = 0.72 cm.) (P = 0.047). However, no differences in the distribution of the calibre of the external carotid artery were found neither by side nor gender. Variations in the level of bifurcation and calibres of carotid arteries are relevant for interventional radiology procedures and head and neck surgeries. Knowledge of these anatomical references might help clinicians in the interpretation of the carotid system.

## Introduction

The variability of the carotid arteries in relation to their diameters and level of bifurcation varied widely in literature^1–5^. The importance of the carotid arteries is based on their distribution territories for face, mouth, eye, nose, brain, on associated pathologies as the atheromatosis and on new diagnostic techniques and interventional approaches^6,7^.

Regarding the descriptions of these arteries, studies were based in different types of sample, founding large samples^3–5,8,9^ or small samples^10–13^ studying different characteristics of the carotid system.

About the carotid bifurcation two landmarks have been described: the upper horn of the thyroid cartilage^5,9^ and the 4th cervical vertebra^14,15^. However, higher levels have been described, such as the hyoid bone, or lower levels, such as the cricoid cartilage^1,5^. In relation with the bifurcation the landmarks have been described at the height of the cervical vertebrae, being exceptionally, in levels as low as C7, Th1 and Th2^16,17^.

The asymmetrical level of carotid bifurcation between the left and right side has also been described^1,7,9,18,19^ even in 48% of the sample size^9^. Variations depending on ethnicity have also been observed, being of a higher level in African Americans than in Caucasians^15^, and in Asian population compared with the Caucasian^20^.

Occasionally, the carotid bifurcation has not been identified in two different situations: one, being associated with an absent common carotid artery, then, the internal and external carotid arteries emerged directly from the brachiocephalic trunk^13,21^; or, on the contrary, associated with an absent external carotid artery, the remaining common-internal carotid artery branches off the cervical arterial branches on its way up the neck^22^.

Regarding the calibre of these arteries, the values obtained a great variability. For instance, it can vary between 5.8 mm and 8.6 mm in the case of the common carotid, depending on the technique with which it has been studied (Conventional Angiography, Computerized Digital Angiography, ECO Doppler, ECO Doppler with colour, Angio-CT and Angio-RMN). Most of the bibliography refers to internal diameters since the radiological techniques made measurements from the margins established by the contrasts used.

A few articles referred to the internal arterial calibre measured from a morphometric analysis^5,23–25^.

Nowadays, the variability reported in relation to the level of bifurcation and calibre has been described in independent studies, not showing these details in a unique article. The samples used in these articles were mostly small and not statistical details were specified in terms of these characteristics. In our study we have unified all variables in a reliable sample with the aim of addressing a simple statistical and useful description for interventional radiology procedures and/or surgeries of head, neck, and face.

## Material and methods

A total sample of 165 embalmed body donors to science belonged to the Department of Anatomy, University of Cambridge, UK, were dissected. The individuals had given their written informed consent prior to death for their use for scientific and educational purposes and donated their bodies. According to National Law, scientific institutions (in general Institutes, Departments or Divisions of Medical Universities) are entitled to receive the body after death mainly by means of a specific legacy, which is a special form of last will and testament. No bequests were accepted without the donor having registered their legacy and been given appropriate information upon which to make a decision based upon written informed consent (policy of ethics); therefore, an ethics committee approval was waived^26^.

These body-donors have been previously dissected by preclinical students and completed by the authors. The gender distribution was 74 male and 91 female body-donors, with an age range of 60 to 103 years. Clinical histories were available, in no case containing any reference to vascular surgical interventions.

Based upon the completeness of the neck structures after dissection, 4 different samples of the available body-donors were taken into account in this study following these parameters: (1) the calibre of the common carotid artery 103 cases (47 male and 55 female); (2) the calibre of the internal carotid artery 139 cases (63 male and 77 female); (3) the calibre of the external carotid artery 206 cases (95 male and 111 female) and (4) the height of carotid bifurcation in 141 cases (61 male and 80 female).

Distances and external diameters were measured with callipers. These measurements were verified by a second investigator. Statistical comparisons were made using the chi-square test, with a value of p < 0.05 taken as statistically significant and the Pearson correlation test. Previous published results were carefully reviewed and compared with this sample.

### Ethical approval

All procedures performed in studies involving human participants were in accordance with the ethical standards of the institutional and/or national research committee and with the 1964 Helsinki declaration and its later amendments or comparable ethical standards.

### Informed consent

Informed consent was obtained from all individual participants included in the study.

According to Spanish and Austrian National Law, scientific institutions (in general Institutes, Departments or Divisions of Medical Universities) are entitled to receive the body after death mainly by means of a specific legacy, which is a special form of last will and testament. No bequests are accepted without the donor having registered their legacy and been given appropriate information upon which to make a decision based upon written informed consent (policy of ethics); therefore, an ethics committee approval is not necessary.

## Results

In order to improve the clarity, the results obtained have been exposed following the order proposed in the "Material and Methods" section.

**Figure 1:**
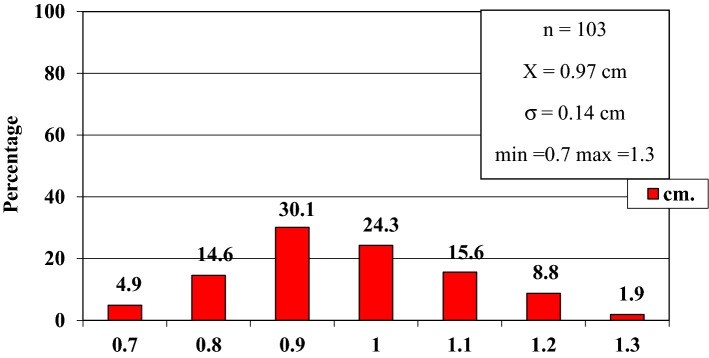
Diameter of the common carotid artery. *cm.* centimetre, *n* sample size, *X* mean, *σ* standard deviation, *min* minimum value and *max* maximum value.

**Figure 2:**
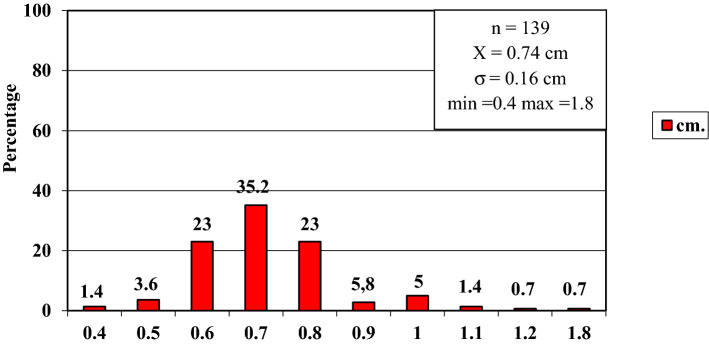
Diameter of the internal carotid artery. *cm.* centimetre, *n* sample size, *X* mean, *σ* standard deviation, *min* minimum value and *max* maximum value.

**Figure 3:**
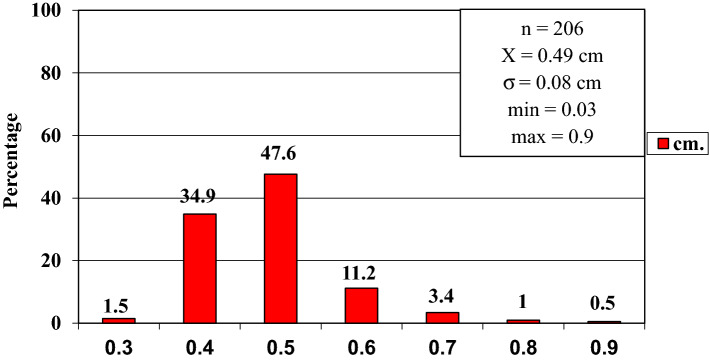
Diameter of the external carotid artery. *cm.* centimetre, *n* sample size, *X* mean, *σ* standard deviation, *min* minimum value and *max* maximum value.

**Figure 4:**
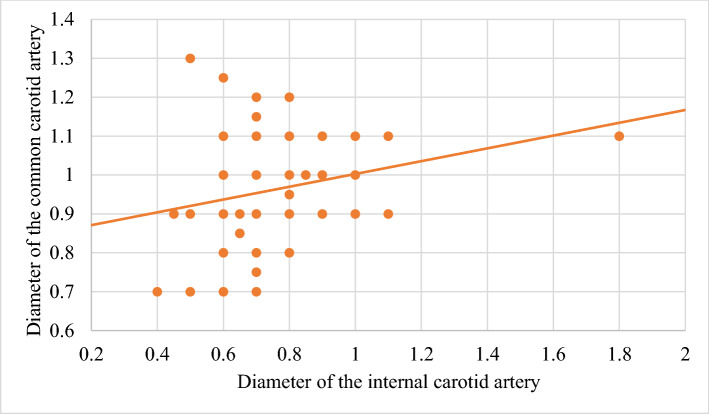
Correlation between the diameters of the common carotid artery and the internal carotid artery.

**Figure 5:**
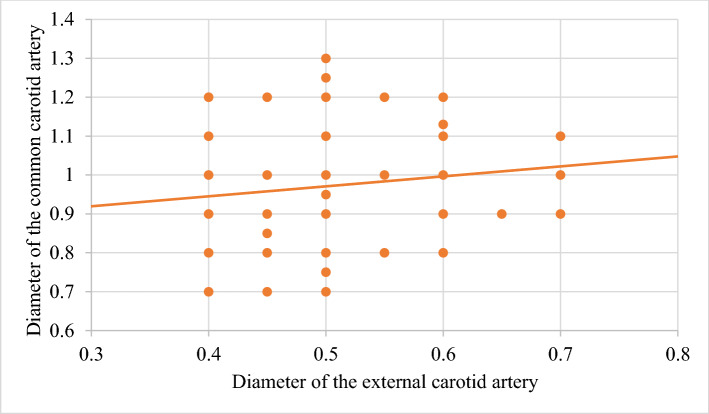
Correlation between the diameters of the common carotid artery and the external carotid artery.

**Figure 6:**
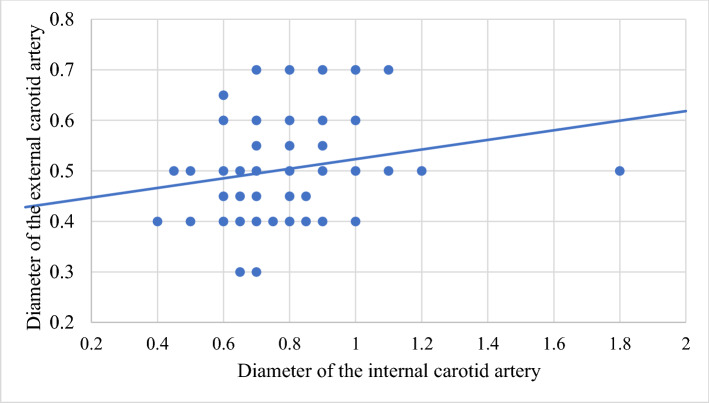
Correlation between the diameters of the internal carotid artery and the external carotid artery.

**Figure 7:**
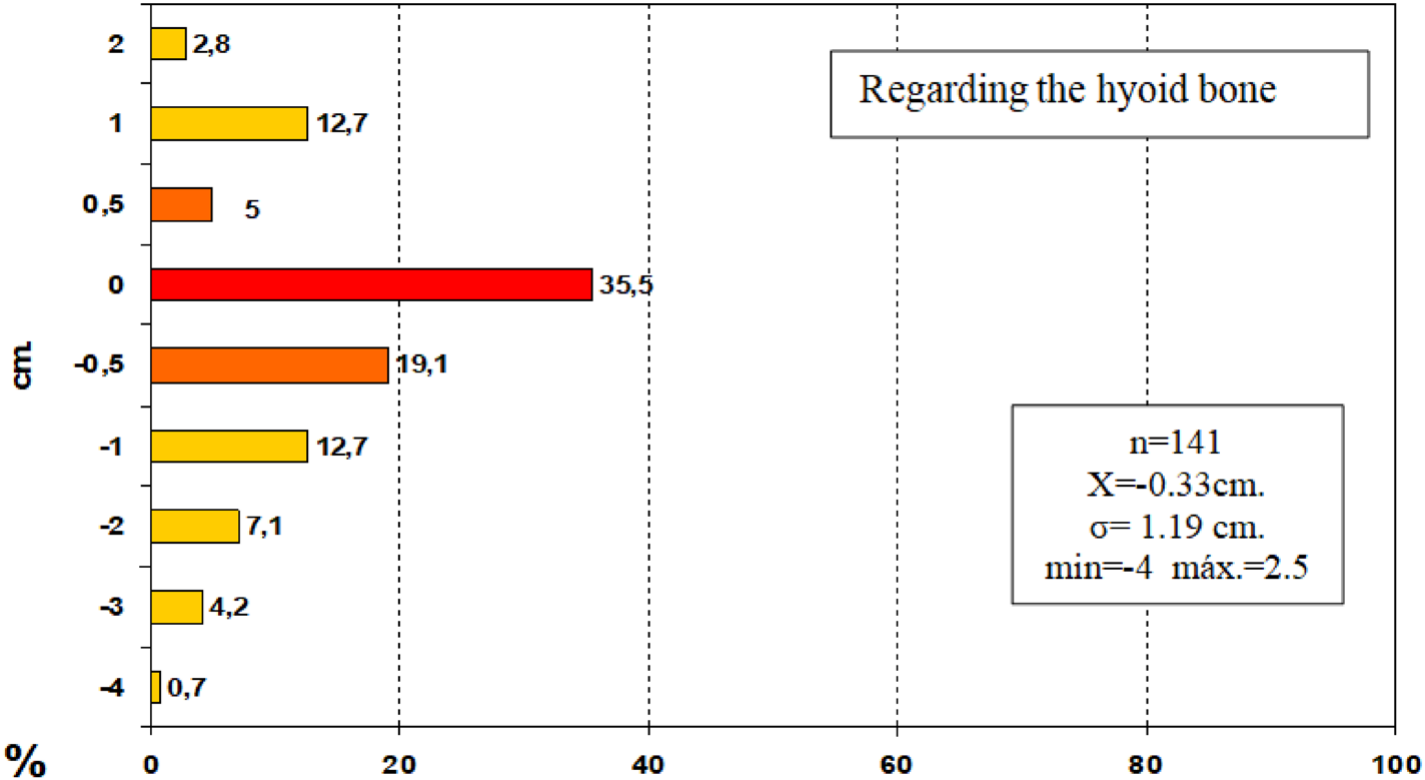
Level of the carotid bifurcation. Negative values are equivalent to heights below the body of the hyoid bone, and positive values are equivalent to heights above the body of the hyoid bone. *cm.* centimetre, *n* sample size, *X* mean, *σ* standard deviation, *min* minimum value and *max* maximum value.

**Figure 8:**
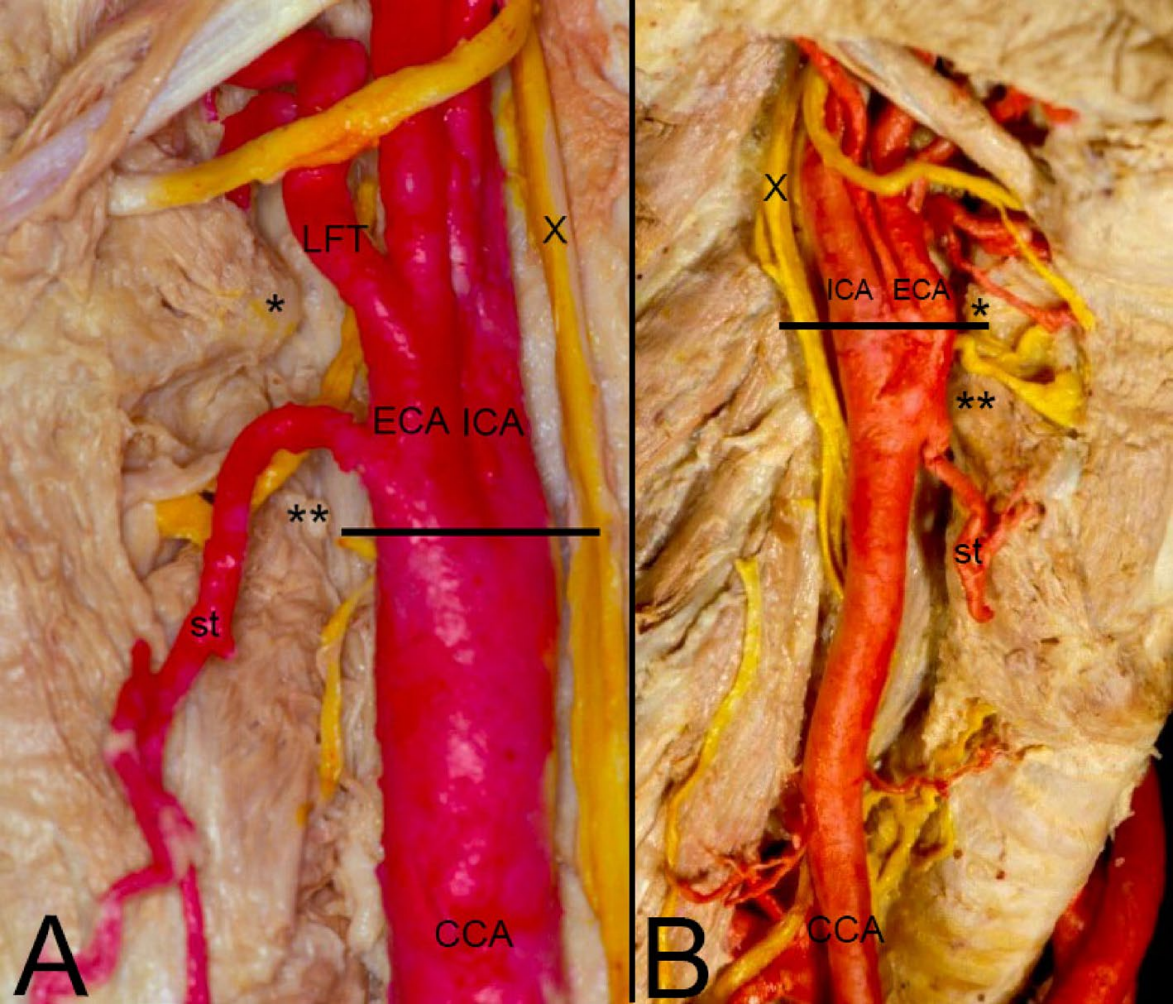
(**A**) Left lateral view of the division of the CCA lowest to the level of the greater horn of the hyoid bone and just on the level of the higher horn of the thyroid cartilage. (**B**) Right lateral view of the division of the CCA just at the level of the greater horn of the hyoid bone. *CCA* common carotid artery, *ICA* internal carotid artery, *ECA* external carotid artery, *X* vagus nerve, *LFT* linguofacial trunk, *st* superior thyroid artery, *Greater horn of the hyoid bone, **Higher horn of the thyroid cartilage, Line: level of the carotid bifurcation.

1. In relation to the calibre of the common carotid artery, it has been studied in a sample made up of 103 cases (47 in male and 56 in female). The values ​​obtained for the calibre of the common carotid artery ranged from 0.7 to 1.3 cm. The average diameter obtained was 0.97 cm with a standard deviation of 0.14 cm (Fig. 1).
  When analyzing the diameter of the common carotid artery depending on the side, no statistically significant differences were observed (p > 0.05). On the contrary, when carrying out the analysis considering the gender variable, these differences have been observed with a significance of p = 0.001. The average arterial calibre of the common carotid artery was higher in male (X = 1.01 cm.) than in female (X = 0.93 cm.).
2. The calibre of the internal carotid artery variable has been studied in a sample made up of 139 cases, 63 in male and 76 in female. The values ​​found for the internal carotid artery ranged from 0.4 cm to 1.8 cm. The average diameter obtained was 0.74 cm. with a standard deviation of 0.16 cm (Fig. 2). When analyzing the diameter of the internal carotid artery according to the side, the diameter of the left internal carotid artery (X = 0.76 cm) was greater than the diameter of the right internal carotid artery (X = 0.72 cm) being statistically significant (p = 0.047). In the case of gender, differences have been observed with a marked tendency towards statistical significance (p = 0.54). The arterial calibre of the internal carotid artery was greater in male (x = 0.77 cm) than in female (x = 0.71 cm).
3. The calibre of the external carotid artery has been studied in a sample consisting of 206 cases, 95 in male and 111 in female. The values ​​found for the external carotid artery ranged from 0.3 to 0.9 cm. The average diameter obtained was 0.49 cm. with a standard deviation of 0.08 cm (Fig. 3.). In the distribution of this arterial calibre by side and gender, statistically significant differences have not been observed (p > 0.05).
  Both the internal and external carotid arteries originate from the common carotid artery, specifically from the carotid bifurcation; therefore, a correlation study has been performed between the diameters of the common carotid artery and the internal carotid artery and between the diameters of the common carotid artery and the external carotid artery.
  The application of the Pearson correlation test has shown that there was no clear correlation between the diameters of the common and internal carotid artery (Fig. 4), but a tendency towards correlation between the diameters of the common carotid artery and the external carotid artery was verified (p = 0.113) (Fig. 5).
  Statistically significant association has been obtained in the correlation between the diameters of the internal and external carotid arteries (p = 0.003) (Fig. 6).
4. In relation to the level of the carotid bifurcation, this variable has been studied in a sample consisting of 141 cases, 61 in male and 80 in female. The level, which has been taken as a reference point, was the body of the hyoid bone. The values ​​obtained for the level of the carotid bifurcation ranged from 4 cm. below the hyoid body and 2.5 cm. above the body of the hyoids. The average height was—0.33 cm., with a standard deviation of 1.19 cm. (Figs. 7, 8A,B). The Klippel-Feil anomaly, typically associated with a low carotid bifurcation, was not found in our sample size.
  In the comparative study between sides, a statistical significance of p = 0.046 has been verified, establishing the right carotid bifurcation at a higher point (x = − 0.19 cm.) than the left carotid bifurcation (x = − 0.48 cm.). On the contrary, no significant differences have been observed in relation to the distribution by gender (p > 0.05).

## Discussion

Most of the references consulted in this work regarding these arteries refer mainly to anatomical variations in relation to their origin, course, or disposition.

Regarding the calibre of these arteries, the results of the studies, as a whole, presented a great variability since they depended on the technique with which it has been studied (Conventional Angiography, Computerized Digital Angiography, ECO Doppler, ECO Doppler with color, Angio-CT and Angio-RMN)^5,23–25^. Furthermore, the diameter measurements with radiological techniques referred to endoluminal diameters since these were made from the margins established by the contrasts used. Only a few articles have been found that referred to the internal arterial calibre measured from a morphometric analysis^5,23,24^ (Table 1).

**Table 1:** Diameter of the carotid arteries.

Calibre	Common carotid artery (CCA)	Internal carotid artery (ICA)	External carotid artery (ECA)
Goubergrits et al., (2002)^24^ n = 86	0.65 cm	0.7 cm	0.46 cm
Ribeiro et al., (2006)^41^ n = 53	Origin: R:0.91 ± 0.02 cm L:0.94 ± 0.02 cm Bifurcation R:1.29 ± 0.04 cm L:1.32 ± 0.04 cm	R: 0.80 ± 0.03 cm L: 0.80 ± 0.02 cm	R:0.73 ± 0.02 cm L:0.71 ± 0.02 cm
Ozgur et al., (2008)^5^ n = 40	Outer diameter of the CCA 2 cm below the CB was 8.1 ± 2.24 mm Outer diameter of CB was 12.79 ± 2.87 mm	Outer diameter of the ICA in proximal was 8.09 ± 2.31 mm Outer diameter of the ICA after carotid sinus was 6.14 ± 1.34 mm	Outer diameter of the ECA in proximal was 6.64 ± 1.32 mm
Our sample, (2020) n = 103	0.97 ± 0.14 cm	0.74 ± 0.16 cm	0.49 ± 0.08 cm

A comparison of our results and those other authors.

*N* sample size, *CCA* Common carotid artery, *ICA* internal carotid artery, *ECA* external carotid artery, *CB* Carotid bifurcation, *M* male, *F* female.

The study by Goubergrits et al.^24^, was carried out from a morphometric analysis with a digital caliper of the luminal diameter of 86 carotid systems that were corroborated with the results of an ECO Doppler study. In our series, these measurements were performed extraluminally with a caliper in a variable sample depending on the artery studied. Comparing both series, a certain similarity has been observed in the diameters of the internal and external carotid arteries, taking into account that the differences obtained could be in relation to the different measurement performed -digital vs. manual- and with the thickness of the arterial wall. However, regarding the difference observed at the level of the common carotid artery—32 mm—these reasons did not justify this huge difference, nor could any reference in relation to the thickness explain it.

In addition, and although it is another artery, the external carotid, in comparison with a radiological study, such as that of Czerwinski et al.^14^ did not obtain such irregular results, since the average diameter obtained by this author for this artery was 0.57 cm (n = 240). Delving into our results with regard to the common carotid artery, this study verified that, with a sample of 103 cases, the values ​​obtained ranged from 0.7 cm and 1.3 cm, and the average diameter was 0.97 cm with a standard deviation of 0.14 cm. Therefore, the obtained values ​​were grouped with little dispersion around the mean value in a sample whose size was greater than 100 cases.

Based on the fact that the external and internal carotid arteries originated from the common carotid artery, a correlation study has been performed between these arteries. No statistically significant correlations between the common and internal carotid arteries and the common and external carotid arteries (p > 0.05) have been observed. Curiously, when studying the correlation between the internal and external carotid arteries, this was established in a statistically significant way (p = 0.003), which together with the results of the previous comparison with another author, could lead us to think about the possibility of a measurement error of the common carotid artery that should be confirmed with a larger sample size in future studies.

The diameter of the carotid arteries has been analyzed in relation to the side and gender with the following conclusions:

- The average diameter of the common carotid artery was higher in male (X = 1.01 cm.) than in female (X = 0.93 cm.) (P = 0.001). We did not observe significant differences regarding the side (p > 0.05).
- The arterial calibre of the internal carotid artery was greater in male (x = 0.77 cm.) than in female (x = 0.71 cm.) With a p very close to statistical significance (p = 0.05). Also, the diameter of the left internal carotid artery (X = 0.76 cm.) was greater than the diameter of the right internal carotid artery (X = 0.72 cm.) (P = 0.047).
- On the contrary, in the distribution of the diameter of the external carotid artery by side and gender, no statistically significant differences have been found (p > 0.05).

In relation to the anatomical level at which the carotid bifurcation occurs, two reference points are classically taken. An anterior one that corresponds to the upper edge of the thyroid cartilage^1,7,12^ and a posterior one, corresponding to the 4th cervical vertebra^3,14^. The upper edge of the thyroid cartilage would be equivalent to the upper edge of the greater horns of the thyroid cartilage^4^.

In most cases the choice of one or the other is in relation to the method chosen when making the measurements. Using a surgical approach, the most common option is the anterior anatomical margin given its greater accessibility. However, when measurements are made using radiological techniques, the most feasible option is the anatomical relief of the vertebral column and bone framework, being, on the other hand, more stable regarding the age variable^3^.

In our case with a study based on body-donor dissection, and an entire sample with an age greater than 65 years, being homogeneous at the age variable, the reference level was the hyoid bone body.

The comparison of our results with those of the consulted bibliography which used a body-donor sample is exposed in Table 2.

**Table 2:** Level of carotid bifurcation studied in cadaveric samples.

Level of carotid bifurcation	Superior to hyoid	Same level as the hyoid	Inferior to the hyoid
Quain, 1844 (n = 285)^7^	3.5%	21%	75.5%
Lucev, 2000 (n = 40)^12^	–	37.5% -Superior level of HB: 12.5% -Inferior level of HB: 25%	62.5% -Superior level of TC: 50% -Inferior level of TC: 12.5%
Lo et al., 2006 (n = 67)^9^	–	55% -Greater horn of HB: 15% - Body of HB: 40%	45% -Superior border of TC: 39% -Body of TC: 6%
Al-Rafiah et al., 2011 (n = 60)^8^	3.3%	25%	66.6% -Between HB and TC: 18.3% -Superior level of TC: 48.3% -Lower than TC: 5%
Mompeó et al., 2015 (n = 38)^18^	–	36.8%	63.1%
Our sample, 2020 (n = 141)	20.5%	35.5%	43.8%

*N* sample size of carotid specimens, *HB* hyoid bone, *TC* thyroid cartilage.

In terms of embryogenesis, the first two aortic arches are commonly lost. However, it could happen that the persistent of the dorsal parts of the second arches becomes the root portion of the stapedial artery on each side or the persistent of connection of the dorsal and ventral parts of the aortic arches give rise to the hypoglossal artery^27,28^. The third and left fourth arches are retained, becoming the root portion of the internal carotid artery and the arcus aortic respectively. On the other hand, both the ventral and dorsal aortae beyond the portion of the third arch are preserved, the former gave rise to the stem of the external carotid directed cranially; some cases of external carotid artery agenesia have been reported^29^. The latter gave rise to the second part of the internal carotid artery, whereas the ventral aorta between the third and fourth arches becomes the stem of the common carotid artery^27^. The movement of the heart downward towards the thorax dragging the carotid arteries could be more or less extensive justifying the different level of division^30^. The asymmetry, right-left, of the blood flow justify differences of caliber observed among the carotid arteries. The collateral and terminal branches of the external carotid artery appear in human embryos of 14 mm to 17 mm by sprouting of the main external carotid trunk^27^. However, there have been reported cases where the collateral arteries arise from the internal or common carotid arteries^10,31,32^.

In our study, the percentage of cases in which the carotid bifurcation occurs at a higher level than the reference level taken previously has been clearly higher (20.5% vs. 3.5% for Quain et al.^7^, 3.3% for Al-Rafiah et al.^8^, and 0% for Lucev et al.^12^, Lo et al.^9^, and Mompeó et al.^18^). Our results have been more similar to those of McAfee et al.^4^, who also observed the possibility of high bifurcations more frequently in a series of 140 hemi sides, since in up to 82% of cases the bifurcation was established in a 2.5 cm area inferior to the branch of the jaw.

In the distribution of the height of the carotid bifurcation according to the side and gender, unlike Smith and Larsen et al.^19^, in our series this height has been established at a higher level on the right side than on the left (p = 0.046) and, unlike Adachi^2^, no differences regarding gender have been observed (p > 0.05).

Therefore, a precise knowledge of possible variation of the carotid axis morphology could help clinicians planning neck or facial surgery (laryngectomy, thyroidectomy), thrombo-endarterectomy, tumor angiogenesis, severe stenosis, aneurysms etc.^33–40^. The surgeon should bear in mind, just before starting any process, the normal, variations and possible anomalies of the carotid axis. These variations and anomalies are present in approximately 1.2% of cases. The normal anatomy, variations or anomalies could be detected easily by imaging techniques as preoperative conventional CT angiography, 3D CT angiography magnetic resonance angiography, digital subtraction angiography, doppler ultrasonography, etc.^33–40^.

The correlation between imaging diagnosis techniques and anatomical studies based on cadaveric samples might be important for accuracy in planning (mini-)invasive carotid techniques. It allows a precise identification of variations or anomalous of the carotid arteries, thereby reducing the possible risk of intraoperative surgery^34,35,40^.

## Conclusions

Variations in the calibres of the carotid arteries (for the common carotid artery 0.97 cm in average, for the internal 0.74 cm in average, and 0.49 cm for the external, respectively) and the carotid bifurcation level (reference point the hyoid bone with an average height of—0.33 cm, right carotid bifurcation at a higher level (x = − 0.19 cm.) than the left one (x = − 0.48 cm.)) are relevant for interventional radiology procedures and head and neck surgeries. A precise knowledge of the anatomical variations could help clinicians in the interpretation of the carotid system allowing them different possibilities in terms of diagnosis and treatment.

Therefore, we should take into account the anatomical variations in the appearance of the carotid system in radio diagnostic images and during surgical approaches, since this variability in the location or size of these structures could have negative consequences for our future patients.

## Data Availability

The datasets generated during and/or analysed during the current study are available from the corresponding author on reasonable request.
